# Development of an mHealth App–Based Intervention for Depressive Rumination (RuminAid): Mixed Methods Focus Group Evaluation

**DOI:** 10.2196/40045

**Published:** 2022-12-13

**Authors:** Eve A Rosenfeld, Cassondra Lyman, John E Roberts

**Affiliations:** 1 Dissemination and Training Division, National Center for PTSD VA Palo Alto Healthcare System Menlo Park, CA United States; 2 Department of Psychiatry and Behavioral Sciences Stanford University Stanford, CA United States; 3 Department of Psychology University of South Florida Tampa, FL United States; 4 Department of Psychology University at Buffalo The State University of New York Buffalo, NY United States

**Keywords:** depression, rumination, mobile health, mHealth, evidence-based treatment, focus group, mental health, mobile app, mobile phone

## Abstract

**Background:**

Depression is a common mental health condition that poses a significant public health burden. Effective treatments for depression exist; however, access to evidence-based care remains limited. Mobile health (mHealth) apps offer an avenue for improving access. However, few mHealth apps are informed by evidence-based treatments and even fewer are empirically evaluated before dissemination. To address this gap, we developed RuminAid, an mHealth app that uses evidence-based treatment components to reduce depression by targeting a single key depressogenic process—rumination.

**Objective:**

The primary objective of this study was to collect qualitative and quantitative feedback that could be used to improve the design of RuminAid before the software development phase.

**Methods:**

We reviewed empirically supported interventions for depression and rumination and used the key aspects of each to create a storyboard version of RuminAid. We distributed an audio-guided presentation of the RuminAid storyboard to 22 individuals for viewing and solicited user feedback on app content, design, and perceived functionality across 7 focus group sessions.

**Results:**

The consumer-rated quality of the storyboard version of RuminAid was in the acceptable to good range. Indeed, most participants reported that they thought RuminAid would be an engaging, functional, and informational app. Likewise, they endorsed overwhelming positive beliefs about the perceived impact of RuminAid; specifically, 96% (21/22) believed that RuminAid will help depressed ruminators with depression and rumination. Nevertheless, the results highlighted the need for improved app aesthetics (eg, a more appealing color scheme and modern design).

**Conclusions:**

Focus group members reported that the quality of information was quite good and had the potential to help adults who struggle with depression and rumination but expressed concern that poor aesthetics would interfere with users’ desire to continue using the app. To address these comments, we hired a graphic designer and redesigned each screen to improve visual appeal. We also removed time gating from the app based on participant feedback and findings from related research. These changes helped elevate RuminAid and informed its initial software build for a pilot trial that focused on evaluating its feasibility and acceptability.

## Introduction

### Background

Depression is the leading cause of global disability [1] and poses a significant public health burden [2-7]. Following the COVID-19 outbreak, approximately one-third of Americans reported clinically significant symptoms of depression [8], highlighting the substantial need for effective treatment. Evidence-based treatments (EBTs) for depression exist; however, access to EBTs remains limited. Less than 35% of individuals with depression receive “minimally adequate” treatment [9-11], and the ratio is even lower for ethnic minority groups [9,10,12,13]. “Stay-at-home” orders during the peak of the COVID-19 pandemic disrupted traditional health care delivery, and our already-overburdened health care system struggled to meet patient needs [14]. Consequently, the pandemic not only increased the prevalence of depression but also reduced treatment accessibility.

Smartphone-based mobile health (mHealth) apps are well suited to address treatment barriers given the relative accessibility of smartphones. In the United States, 85% of adults own a smartphone, and 91% of users report that their smartphone is within arm’s reach 24 hours per day [15]. Consequently, mHealth apps can be accessed at any time, allowing for real-time intervention. In addition, 15% of Americans reported that their only internet-accessible device was their smartphone, and marginalized communities reported higher proportions of smartphone-only internet access [16]. Furthermore, emerging mHealth interventions—particularly self-guided interventions—alleviate the burden on the mental health care system by providing contactless, automated interventions and facilitating self-management.

Unfortunately, many mHealth apps purported to treat depression are not informed by evidence-based care. Specifically, only 10% of publicly available depression apps use evidence-based principles of cognitive behavioral therapy or behavioral activation (BA) [17]. Of those that are empirically informed (eg, MoodTools [18]), few have been empirically evaluated. A systematic review found that only 2% of publicly available mental health apps were empirically supported [19]. Thus, it is unclear to what extent specific mHealth apps are efficacious or effective treatments for depression.

A minority of depression apps have been informed by EBTs and subjected to at least an initial empirical evaluation. For example, Moodivate is a BA self-help app involving psychoeducation, identification of personal values, activity scheduling, and daily mood ratings [20]. A pilot trial found initial evidence of its feasibility and efficacy [21], and a large-scale randomized clinical trial is underway [22]. Nevertheless, too few app-based interventions are subjected to rigorous empirical testing before they reach the market.

In addition, existing evidence-informed mHealth apps, such as Moodivate, target depressive symptoms by delivering an app version of a traditional face-to-face psychotherapy protocol using a varied set of nonoverlapping skills (eg, values clarification, cognitive restructuring, and activity scheduling). These tend to be presented in a nonstepwise fashion, with all psychoeducational material available at the outset in a handout format. This approach is inconsistent with how consumers use apps: in frequent, short bursts [23]. Treatment engagement may suffer if mHealth interventions require lengthy engagement, are overly didactic, or lack a clear sequential structure. Therefore, we argue that it might be most useful for an mHealth app to target a specific psychological process using a limited set of scaffolded skills [23]. Each targeted skill builds on the skills previously developed as the user progresses through the app content. The intervention would include minimal, targeted psychoeducation and narrowly focus on brief, sequential skill-oriented tasks that are “gamified.”

To date, no empirically supported mHealth apps have focused on rumination, despite it being a potentially promising treatment target. According to response styles theory [24], rumination is a pattern of behaviors and cognitions that focus attention on depressive symptoms, including their causes, consequences, and implications [25]. Rumination causes excessive focus on negative emotional states, inhibits mood-enhancing behaviors, and exacerbates and prolongs depression [24,25]. Although various theories of rumination conceptualize its content, antecedents, and functions somewhat differently [26,27], most converge on key features: rumination is a specific, depression-related form of repetitive negative thinking that occurs in response to a triggering event, and it is experienced as distressing and difficult to control [28-30].

Overwhelming evidence demonstrates a strong relationship between rumination and depression. Rumination prospectively predicts higher levels of depressive symptoms over time [31-33], is a trait of vulnerability to depression [34-37], contributes to the maintenance of depressive episodes [24,25], is a precursor to the onset of clinical depression [32,35,37,38], and is a risk factor for relapse and recurrence [39-42]. Experimental studies have causally linked rumination to the maintenance of depression [25,43,44] and have demonstrated its co-occurrence with other forms of psychopathology [45-52]. Rumination functions as an experiential avoidance strategy (ie, avoidance of uncomfortable private experiences [53]) wherein ruminators distract themselves from emotionally arousing material (eg, sadness) through repetitive thinking, which contributes to negative sequelae [54].

Several EBTs explicitly target rumination, including BA, mindfulness-based cognitive therapy, and rumination-focused cognitive behavioral therapy (RFCBT). Although these interventions are well supported by research [55-57], they are neither widely disseminated nor easily accessible. Considering that depression is so pervasive and problematic, rumination is strongly implicated in depression, and effective treatments for rumination exist but are not widely disseminated, rumination is a promising target for mHealth intervention. To address the need for an easily accessible evidence-based intervention targeting rumination, we designed RuminAid—a new mHealth app.

### Treatment Development and Description

#### Overview

RuminAid skills were drawn from well-supported EBTs for depression and rumination (eg, BA, mindfulness-based cognitive therapy, and RFCBT) and mHealth research, which highlighted the importance of balancing EBT components with simplicity and user friendliness by distilling EBTs to key components. We identified five essential components: (1) brief psychoeducation, (2) recognizing ruminative episodes, (3) alternative behaviors to replace rumination, (4) counteracting rumination-related attentional deficits, and (5) gamification.

RuminAid integrates these elements across 5 brief lessons presented to users as sequential “quests,” with users completing 1 quest per day. Each quest includes brief psychoeducation and gamified elements. Quests 1 to 2 focus on identifying rumination. Quests 3 to 4 teach users to use alternative behaviors to combat rumination. Quest 5 teaches users mindfulness and behavioral skills to counteract the deleterious effects of rumination on attention.

#### Brief Psychoeducation

Brief psychoeducation is integrated into each quest (Multimedia Appendix 1). This material identifies rumination as a treatment target, normalizes rumination as an experience, addresses harmful meta-cognitive beliefs, and orients users to new skills. As overemphasis on didactic content is inconsistent with typical smartphone use [23], psychoeducation is not the primary focus. Instead, psychoeducation is intended to facilitate skills acquisition and is limited to a few minutes per quest, at the most.

#### Identifying Rumination

RuminAid teaches users to discriminate between rumination and nonpathological processes (eg, problem-solving and introspection) and familiarizes users with rumination warning signs (Multimedia Appendix 2). For example, RuminAid users explicitly label and log periods of rumination in real time, tracking associated content, triggers, and internal contexts (eg, thoughts and emotions). The “Two-Minute Rule for Recognizing Rumination” involves engaging in current patterns of thinking for 2 minutes and then answering specific questions to determine if the user is ruminating [58].

To accommodate the idiographic nature of rumination, users store their ruminative content, triggers, and contexts in a personalized, editable list of rumination “red flags” (ie, signs they are ruminating). RuminAid also includes a list of “common red flags” that users can save on their personalized list. When logging rumination, items from the personalized lists of users are available for tracking via a drop-down menu. Moreover, users can use text entries to enter novel red flags. As users familiarize themselves with their personal red flags, they learn to quickly identify and label periods of rumination, allowing for rapid application of later therapeutic skills.

#### Alternative Behaviors

RuminAid is behaviorally oriented, providing users with specific alternative behaviors to replace unhelpful rumination habits [59,60] and counteract its avoidance function [61]. RuminAid includes 2 forms of alternative behaviors: self-soothing and approach-oriented behaviors (Multimedia Appendix 3). Both forms of alternative behaviors can be conceptualized as BA strategies, wherein self-soothing behaviors aim to increase pleasure and improve mood, whereas approach behaviors aim to increase mastery and promote problem-solving over avoidance.

Self-soothing behaviors provide distressed individuals with a sense of calmness or pleasure and are promoted in EBTs (eg, BA [57] and Dialectical Behavior Therapy [62,63]). In quest 3, users are instructed to engage in self-soothing whenever they ruminate and are automatically prompted to do so every time rumination is logged. Self-soothing helps disrupt the ruminative cycle and replace the rumination-avoidance association with a rumination-action association. Self-soothing orients users to use “rumination as a call to action,” preparing them for the greater challenge of implementing approach-oriented behaviors. Practicing self-soothing (rather than avoidance) introduces opportunities for positive reinforcement, potentially improving mood. Users are briefed on the distinction between avoidance and self-soothing behaviors and how to discriminate between them (Multimedia Appendix 4). Specifically, RuminAid emphasizes observing the functional consequences of a behavior on one’s mood and thoughts. If a given behavior results in improvements in mood and disrupts the ruminative thought cycle, it can be used again as an effective self-soothing behavior. In contrast, if a given behavior results in emotional numbness and temporary distraction from ruminative thoughts, the behavior is not an effective self-soothing behavior. Users are also taught that the same behavior (eg, watching a comedy show) might function as an effective self-soothing behavior for one person but as an avoidance behavior for another person. Users are encouraged to try new self-soothing behaviors to explore which options work best for them. They are able to edit their list of self-soothing behaviors at any time.

The second category of alternative behaviors is approach-oriented behaviors. Introduced in quest 4, approach-oriented behaviors are aimed at directly counteracting avoidance. Specifically, RuminAid helps users to create a new, adaptive habitual response to rumination and its triggers, a strategy drawn from EBTs (eg, BA and RFCBT). Users are taught to identify what they are avoiding during rumination by using their rumination topics. They are then instructed to generate and engage in alternative approach-oriented behaviors. These behaviors focus on addressing avoidance head-on and are framed as “facing your fears” (akin to exposure) or “doing the opposite” of the avoidance impulse (akin to opposite action in dialectical behavior therapy). Examples are provided to users to demonstrate these concepts (Multimedia Appendix 5). In addition, users are provided with tools to identify when self-soothing or approach-oriented behavior techniques may be more helpful in a given context.

#### Counteracting Rumination-Related Attentionionl Deficits

Rumination has negative effects on attention and concentration, particularly attention-switching [64-69], that is, the ability to flexibly attend to and adjust behavior in accordance with changes in task goals [70]. Resource allocation theory [71-73] suggests that depression-related thoughts consume cognitive resources, making it difficult for ruminators to attend to task-relevant processes [74]. Because of this, ruminators may not be fully attentive to their experiences while executing alternative behaviors, which could reduce purported positive effects. RuminAid teaches users to use mindfulness (ie, purposeful, present-focused, and nonjudgmental awareness of internal experiences and external environment; Multimedia Appendix 6) to counteract these deficits during quest 5. This approach has effectively reduced rumination in other interventions [75-77]. RuminAid users are taught to choose active rather than passive alternative behaviors and to enhance present-moment awareness using the 5 senses. RuminAid also includes formal mindfulness exercises that can be used as alternative behaviors.

#### Gamification

RuminAid rewards treatment engagement through gamification (Multimedia Appendix 7). Gamification enhances user experience in mHealth apps by integrating gaming elements (eg, completing a quest map) into the intervention [19]. Although gamification research is limited, initial evidence has suggested that gamified elements reduce attrition and increase engagement by creating enjoyable, engaging, and reinforcing experiences [19,78,79]. Pleasurable gaming experiences trigger dopamine and endorphin release [80]. By triggering this response, game-like experiences in digital interventions may reinforce engagement [81]. A meta-analysis found moderate effect sizes for the effectiveness of gamified digital depression interventions [81].

RuminAid’s quest structure and “map” gamifies psychoeducation and assignments. Progressing in a given quest is rewarded by earning a corresponding star on the map, unlocking new app features and map stages, and prompting celebratory messages of encouragement. These elements should facilitate more active and enjoyable user experiences and reinforce engagement.

### Goal of This Study

An overwhelming number of mHealth apps claim to treat depression, but a minority are empirically supported [19]. To address this issue directly, we have and will continue to incorporate scientific inquiry into RuminAid from development to dissemination in an iterative, data-driven process. This study aimed to estimate the initial acceptability of RuminAid by evaluating its user-perceived quality and identifying the potential modifications required. We distributed a storyboard presentation of the initial version of RuminAid to potential consumers and collected quantitative and qualitative feedback via individual surveys and focus group interviews. This feedback was used to facilitate improvements in RuminAid before testing its feasibility, acceptability, and effectiveness.

## Methods

### Participants

We recruited participants from the University at Buffalo Psychology 101 courses. Individuals were eligible to participate if they were native English speakers and adults (age ≥ 18 years). Participants were oversampled for moderate, moderately severe, or severe depression, but individuals with lower depression scores were also allowed to participate. A total of 22 individuals completed all the required surveys, viewed the complete storyboard presentation, passed the attention check items, and attended a focus group interview. We conducted a total of 7 focus group sessions with groups ranging in size from 1 to 7 participants (mean 3.14, SD 2.04; median 3).

Our sample comprised 77% (17/22) men and 23% (5/22) women. The mean age of the participants was approximately 19 (mean 18.86, SD 0.83) years. The sample was primarily heterosexual (19/22, 86%), and 14% (3/22) of the participants were bisexual. None of the participants endorsed any other sexual orientation. The sample consisted mainly of first- and second-year students: 68% (15/22) were first-year students, 23% (5/22) were second-year students, and 9% (2/22) were third-year students. In terms of marital status, most (20/22, 91%) participants were single and a minority (2/22, 9%) were married or partnered. In terms of religious background, 46% (10/22) identified as Catholic, 9% (2/22) as Protestant, 5% (1/22) as Muslim, and 9% (2/22) as some other religion (Christian nondenominational, n=1; spiritual, n=1), whereas 32% (7/22) of the participants reported that they did not identify with a religion. Participants were allowed to select all ethnic and racial identities with which they identified. The ethnic and racial makeup of the sample was as follows: 50% (11/22) White, 36% (8/22) Black, 18% (4/22) Latin American, and 9% (2/22) Asian American and Pacific Islander.

Regarding depressive symptoms, 41% (9/22) of the participants reported minimal symptoms of depression based on the Patient Health Questionnaire (PHQ; PHQ-8), 41% (9/22) reported mild depression, 9% (2/22) reported moderate depression, and 9% (2/22) reported moderately severe depression. None of the participants reported experiencing severe depression. The participants had an average PHQ-8 score of 6.55 (SD 5.12) and a median score of 5, which indicated that, overall, the participants experienced mild depression.

### Measures

#### PHQ-8 Measure

The PHQ-8 [82-84] is an 8-item self-report measure of depressive symptoms and severity. Items are rated on a 4-point scale, with higher scores indicating more severe depression. The PHQ-8 omits the self-harm item from the PHQ-9 and is often used in research settings where interventions for suicidality or self-injury are difficult to coordinate [83]. The PHQ-8 has demonstrated reliability and validity as a measure of depression severity [83,84]. The PHQ-8 was used to oversample for moderate or worse depression (PHQ-8 score ≥10) at screening and readministered at baseline. Scores at readministration were used for all analyses.

#### Mobile Application Rating Scale: User Version

The Mobile Application Rating Scale–user version (uMARS [85]) is a 26-item measure of user-rated app quality consisting of the following scales: (1) engagement (degree of fun, interestingness, customizability, interactivity, whether it has prompts such as sending alerts, reminders, etc), (2) functionality (app functioning, ease of use, navigation, flow logic, and gestural design), (3) aesthetics (graphic design, visual appeal, color scheme, and stylistic consistency), (4) information (contains high-quality information from a credible source), (5) app quality (average mean score of the 4 preceding scales), (6) subjective app quality (overall like or dislike), and (7) perceived impact (whether this app will help the target population with the target problem). Subjective app quality and perceived impact scales can be reported as individual items or mean scores. For our purposes, we used mean scores. To identify qualitative descriptors for each scale (1=inadequate, 2=poor, 3=acceptable, 4=good, and 5=excellent), the average mean scores were rounded to the nearest whole number [86]. The uMARS has demonstrated good test-retest reliability and excellent internal consistency [85].

#### Focus Group Interview

The semistructured focus group interview was designed specifically for this study to collect qualitative feedback through a standardized set of questions. Initial questions asked participants to elaborate on uMARS ratings and identify potential improvements for items rated <4 by at least one focus group member. Additional questions included, “what app features did you find most helpful?” and “based on your experience trying out RuminAid, do you think this app would help you to identify rumination in your day-to-day life?” The interviews lasted for 1 hour and were moderated by the first author through Zoom videoconferencing (Zoom Video Communications).

Qualitative content analysis [87] was used to systematically identify and describe themes within the participants’ focus group responses. Main categories were generated in a content-driven manner (ie, using theoretical models to derive categories [87]) derived from uMARS scales—engagement, functionality, aesthetics, information, subjective quality, and perceived impact; we added an “other” category for feedback that fell outside of these domains. Subcategories within these domains were generated in a data-driven manner (ie, using the data collected to derive categories [87]). We used subsummation (ie, an iterative process whereby relevant concepts were identified, compared with existing categories, added to existing categories if appropriate, or used to define new categories [87]) to add data-driven subcategories until saturation (ie, the inability to find additional concepts [87]) was achieved. For example, we identified that participants tended to comment on the entertainment value of the app, whether entertainment would impact engagement, and ideas they had about how a social media component could be added to the app to increase engagement. We created 3 corresponding data-driven subcategories within the engagement domain: “Not Entertaining,” “Entertainment not Important,” and “Social Media.”

Transcriptions of the recorded interviews were coded by 2 trained research assistants for the presence or absence of positive and negative statements on each content domain. “Positive statements” were operationalized as comments that were positive in nature, such as liking elements of the app, suggesting that existing elements be retained, recommendations to further capitalize on well-liked components, or explicit agreement with another participant’s positive statement. “Negative statements” were operationalized as comments that were negative in nature, such as suggesting modifications to a disliked element, recommending the removal of elements, or explicit agreement with another participant’s negative statement. Within a given domain, positive and negative statements were not mutually exclusive, that is, an individual participant could have made both positive and negative statements. Responses were coded according to the presence or absence of statements related to each subcategory.

### Procedures

Participants were screened through mass testing using the University at Buffalo’s Sona Systems to determine whether they met the eligibility criteria. Eligible participants signed up for this study on the University at Buffalo’s Sona Systems and attended a web-based focus group session. The sessions were conducted on a web-based videoconferencing platform (Zoom). Upon enrollment, the participants completed a demographics questionnaire and the PHQ-8 via Qualtrics. Next, participants were provided with a link to view the RuminAid storyboard using Panopto, which is a lecture recording and streaming platform. Participants’ progress in viewing and listening to the presentation was tracked using Panopto, which allowed study staff to view the percentage of the presentation the participants had completed. The participants had to complete 100% of the storyboard to proceed with the study. After viewing the storyboard presentation for 2 hours, 2 minutes, and 53 seconds, participants completed the follow-up Qualtrics survey, which consisted of the uMARS and attention-check questions that the participants needed to answer correctly to participate in the focus groups. The correct answers to the attention-check items were embedded within the audio of the storyboard presentation, with clear instructions to make a note of the information for the attention-check items. This ensured that the attention-check items would be easy to answer correctly for participants who attended to the presentation and quite difficult for those who did not. Next, participants attended a scheduled focus group session and provided feedback in a discussion-based format. Focus group sessions were video- and audio-recorded and then transcribed and coded.

### Ethical Considerations

The University at Buffalo Institutional Review Board deemed this project exempt from review; all documents used were reviewed and approved by the institutional review board. Before participating, participants were informed that this research was being conducted to investigate the user-perceived quality of a new mHealth smartphone app called RuminAid and that their feedback would be used to improve the quality of the app. We told participants that their responses would be deidentified and stored on a password-protected server. We did not collect identifying information (eg, name, email, and phone number), but participants could provide their email address to opt in to receive a free download of RuminAid once it reached the marketplace; this was entirely optional. Similarly, participants could opt out of their feedback being anonymously quoted in publications about RuminAid. Participants were told that they had the right to end the study at any time and were compensated with credits that partially fulfilled the course research requirement.

## Results

### Quantitative Feedback

In our sample, engagement (mean 3.58, SD 0.65), functionality (mean 3.86, SD 0.64), information (mean 4.07, SD 0.46), app quality (mean 3.63, SD 0.47), and perceived impact (mean 3.63, SD 0.47) were rated as “good.” In contrast, aesthetics (mean 3.02, SD 0.66) and subjective app quality (mean 2.89, SD 0.64) were rated as “acceptable.”

### Qualitative Feedback

#### Engagement

During focus group sessions, 50% (11/22) of the participants made positive statements about engagement (ie, degree of fun, interestingness, customizability, interactivity, whether it has prompts such as sending alerts, reminders, etc) and 86% (19/22) made negative statements about engagement. This suggested potentially mixed feelings about how engaging RuminAid was for the participants. Within this domain, 64% (14/22) of the participants reported that they did not find the app entertaining. However, 36% (8/22) of the participants felt that the entertainment value of the app was unimportant, given that its purpose was related to mental health, not entertainment. In addition, 18% (4/22) of the participants suggested adding a social media element to RuminAid to improve engagement.

#### Functionality

In terms of app functionality (ie, app functioning, ease of use, navigation, flow logic, and gestural design), 55% (12/22) of the participants made positive statements and 41% (9/22) made negative statements. This suggested that there were mixed feelings about app functionality, although most participants identified positive aspects of app functionality. Specifically, 27% (6/22) of the participants noted that RuminAid might be difficult to navigate. In contrast, 50% (11/22) of the participants reported that the flow logic of the app (ie, how the content progresses from one screen to the next) made sense.

#### Aesthetics

Regarding aesthetics (ie, graphic design, visual appeal, color scheme, and stylistic consistency), 9% (2/22) of the participants made positive statements, whereas 96% (21/22) of the participants made negative statements. This suggested that app aesthetics was a major concern for the participants. Specifically, 91% (20/22) of the participants described the color scheme of RuminAid as boring, “drab,” “depressing,” or dull. In addition, 18% (4/22) of the participants expressed that this issue would interfere with app use and decrease the likelihood of initial or continued app use, although 5% (1/22) of the participants stated that the color scheme would not interfere. In terms of graphics, 45% (10/22) of the participants expressed that these appeared amateur and 59% (13/22) stated that the graphics appeared outdated.

#### Information

Participants also commented on the information included in RuminAid (ie, containing high-quality information from a credible source): 77% (17/22) of the participants made positive statements and 32% (7/22) made negative statements. This indicated that, overall, information appeared to be a relative strength of the app. More specifically, 73% (16/22) of the participants stated that the amount of detail included in the information was appropriate and to their liking. A minority (4/22, 18%) of the participants found that there was excessive detail. None of the participants reported insufficient detail. Furthermore, 59% (13/22) of the participants reported learning something new from RuminAid. In terms of credibility, 18% (4/22) of the participants explicitly reported feeling that information came from a credible source; however, 23% (5/22) of the participants felt that credibility could be improved if the sources were cited within the app.

#### Subjective Quality

The subjective quality of the app (ie, overall like or dislike of the app) was overwhelmingly positive: 96% (21/22) of the participants made positive statements about the subjective quality of RuminAid. None of the participants made negative statements regarding the subjective app quality. This suggested that the participants felt positive about the app as a whole.

#### Perceived Impact

Participants endorsed overwhelmingly positive beliefs about the perceived impact of RuminAid: 96% (21/22) of the participants indicated that they believed RuminAid would help depressed ruminators with their depression and rumination. None of the participants made negative statements regarding the perceived impact of RuminAid. This indicated that the participants saw the app as potentially effective and helpful for depression and rumination.

#### Other

Approximately 18% (4/22) of the participants made positive statements and 36% (8/22) made negative statements that did not fit within the aforementioned domains. Specifically, 46% (10/22) of the participants reported that integration of measurement-based care features would improve the app, and 23% (5/22) of the participants raised concerns that “time gating” (ie, when users are prevented from accessing new app content until a certain amount of time has passed) quests with a 1-week delay between quests (as originally planned) would negatively impact app quality, frustrate users, and decrease engagement.

**Figure 1:**
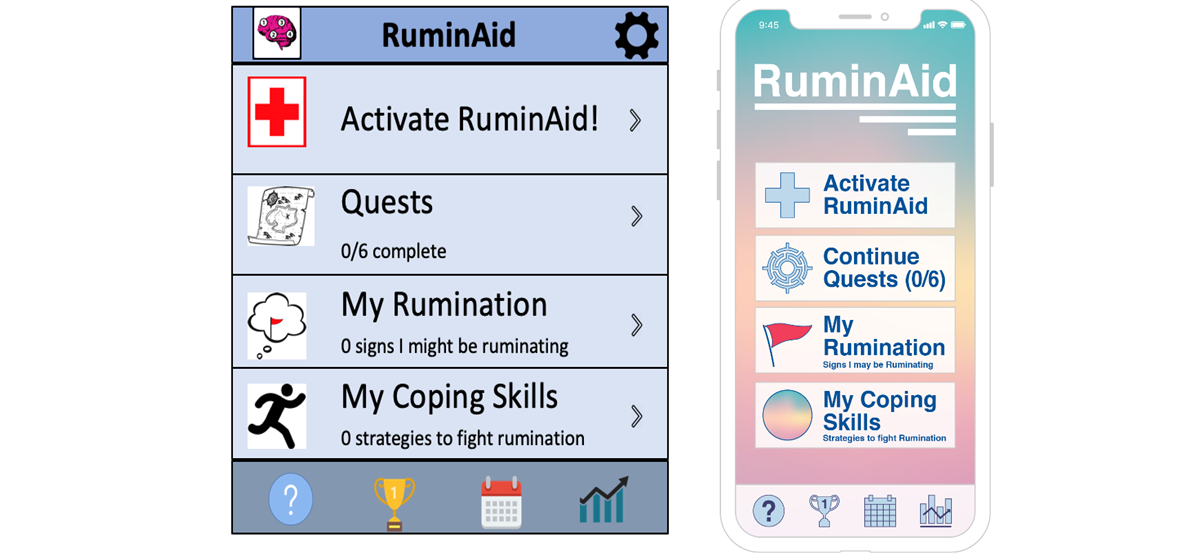
Home screen redesign. RuminAid home screen before user-centered redesign (left) and after user-centered redesign (right).

**Figure 2:**
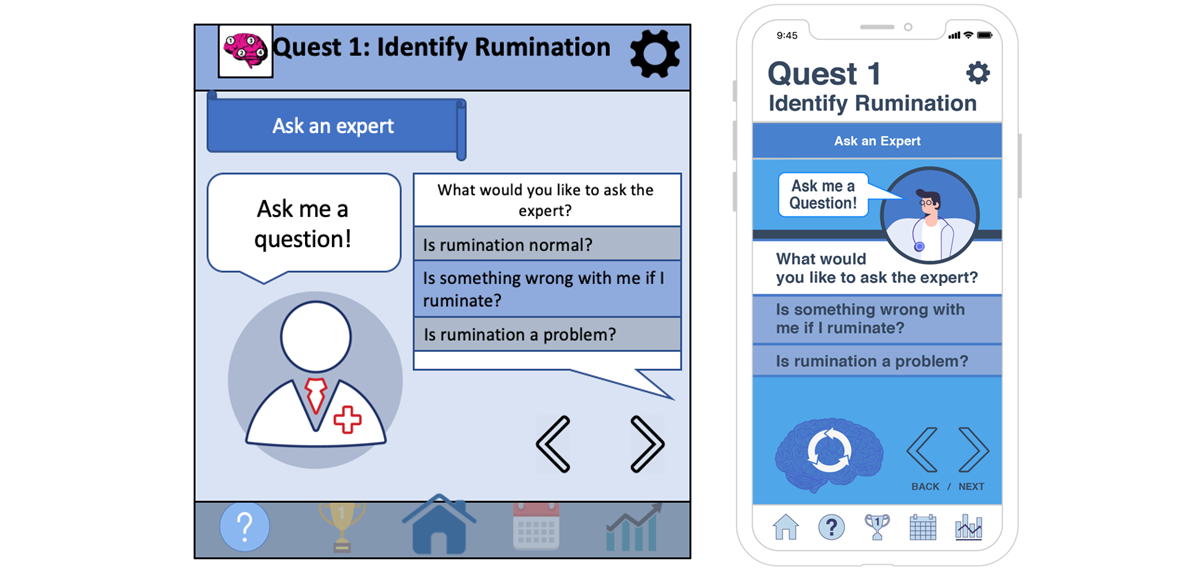
Ask an expert screen redesign. RuminAid’s first set of “Ask an Expert” screens before user-centered redesign (left) and after user-centered redesign (right).

**Figure 3:**
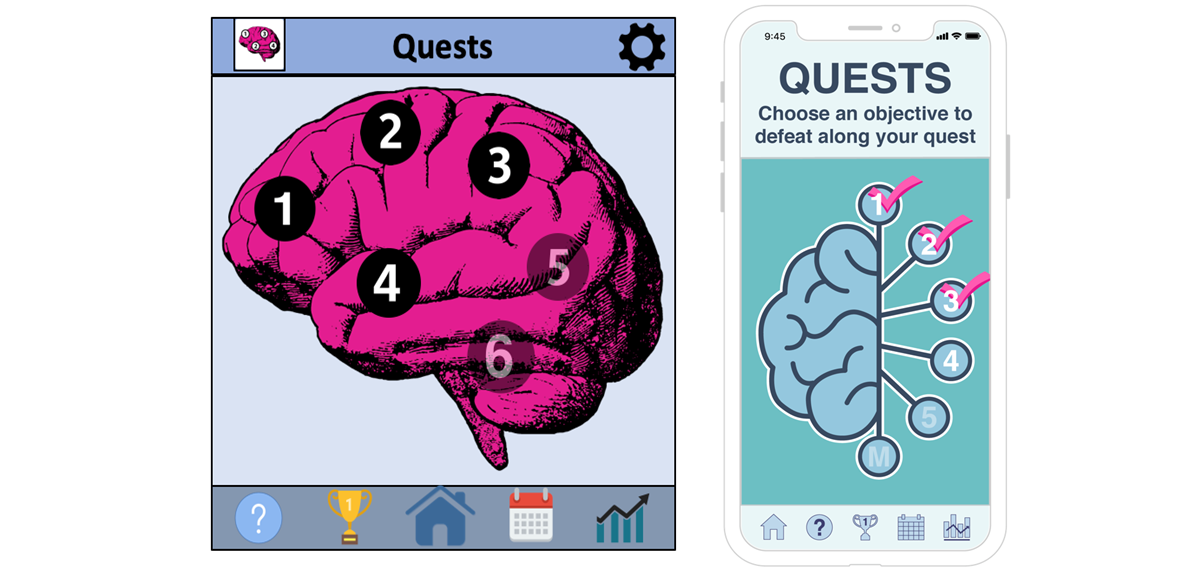
Quest launch screen redesign. Quest 4 launch screen before user-centered redesign (left) and after user-centered redesign (right).

Example quotes from participants and the interrater reliability for each domain and subcategory mentioned above are included in Tables 1 and 2. Examples of modifications based on focus group feedback can be seen in Figures 1-3.

**Table 1:** Domains of qualitative focus group feedback^a^.

Domain			Description		Quotes from participants		Cohen κ^b^
**Engagement**							
	Positive	App is fun, interesting, customizable, or interactive and has prompts (eg, sends alerts, messages, reminders, or feedback or enables sharing)		“I feel like [engagement] is kind of the whole point. And I think that it does it pretty well.” “I really like games. So, going through the levels—I really liked that and how it was kind of an incentive to keep going.”		0.91	
	Negative	App is not fun, interesting, customizable, or interactive or does not have prompts (eg, sends alerts, messages, reminders, or feedback or enables sharing)		“I would say it could be a little more interactive, because the steps are just so repetitive and it’s kind of the same thing going through each task.”		1	
**Functionality**							
	Positive	App functioning well; easy to learn; or good navigation, flow logic, or gestural design		“I thought the format where everything is laid out, like, the layout is good.” “My favorite part of the app personally was the quest system put in place.”		0.47	
	Negative	App not functioning well; not easy to learn; or poor navigation, flow logic, and gestural design		“[You should] make the flow a little smoother.”		0.38	
**Aesthetics**							
	Positive	Graphic design, overall visual appeal, color scheme, and stylistic consistency are good or appealing		“The brain graphic...was pretty good with the pink on blue brain contrast.”		0.8	
	Negative	Graphic design, overall visual appeal, color scheme, and stylistic consistency are not good or unappealing		“I feel like a complete artistic overhaul of the app needs to be done.”		0.38	
**Information**							
	Positive	App contains high-quality information (eg, text, feedback, measures, or references) from a credible source		“It had a lot of solid information that would be useful to someone struggling with depression.”		1	
	Negative	App does not contain high-quality information (eg, text, feedback, measures, and references) or lacks credible source		“Some of the information seems sort of redundant.”		0.51	
**Subjective quality**							
	Positive	Overall positive impression or liked the app		“Everything that was presented was presented clearly. So, I was able to retain it better, and actually learn about it...I also like some of the smaller, finer details...certain ways of rewarding you for staying on the app.”		1	
	Negative	Overall negative impression or did not like the app		No participants made negative comments about subjective quality.		1	
**Perceived impact**							
	Positive	This app will help the target population (ie, depressed ruminators) with the problem (ie, depression and rumination)		“I think it would help because it does have those red flag areas, you make it customizable for you...I think that it would definitely be helpful for people.” “If I were struggling with rumination and depression, it would be eye-opening in a way...If you didn’t realize you were doing that, or how it affected you, you’re going to get a new perspective to see why it’s affecting you and how you can stop ruminating.”		1	
	Negative	This app will not help the target population (ie, depressed ruminators) with the problem (ie, depression and rumination)		No participants made negative comments about perceived impact.		1	
**Other**							
	Positive	Additional themes that emerged but did not fit into the above categories; positive valence to this feedback		“When I first started looking at it, I thought it was going to be too short...I like how it’s a process, because at first I almost wrote it off. I felt like it was kind of just like, ‘this is a quick fix’ type thing. But a lot of mental health things aren’t a quick fix. So, I like how it was a process that you go through and you can plan out ahead of time and that’s how you can really make a change with it is by making it a process.”		0.31	
	Negative	Additional themes that emerged but did not fit into the above categories; negative valence to this feedback		“I would say maybe like further on down the road, make sure you keep updating it, because if someone uses it frequently, they could very easily go through all quests very quick. And then after that, there’s really no use for it.”		0.36	

^a^Descriptions and examples of categories identified in qualitative focus group feedback.

^b^Cohen κ represents an estimate of interrater reliability for qualitative items.

**Table 2:** Subcategories of qualitative focus group feedback^a^.

Subcategories			Description		Quotes from participants		Cohen κ^b^
**Engagement**							
	Not entertaining	The app was not entertaining		“I saw a lot of stick figure-like images. And if they were just replaced with humans or something doing the same thing, it would have been more entertaining.”		0.8	
	Entertainment not important	The entertainment value is not relevant to this app, is not an important factor, or is not the purpose of the app and would not impact the likelihood of using it		“I don’t really feel like it’s intended to be entertaining. It’s meant to help somebody.” “I wouldn’t, at least for me, you know, as somebody that would be downloading it, I don’t consider the entertainment aspect necessarily a priority.”		0.56	
	Social media	Adding a social media component to the app would make it more entertaining or engaging		“I would [add] a feature where you can connect with other users and maybe talk about with another actual person, like what’s going on, and they can give you like feedback or something like that.”		1	
**Functionality**							
	Difficult navigation	The app appears difficult to navigate		“I felt like...it jumped around a little bit. [I suggest] making the flow of it better.”		0.38	
	Logical flow	The app flow makes sense		“I liked how it was kind of structured so that you aren’t just having a bunch of information dumped on you, it’s kind of separated into these five larger segments, that you slowly make your way through with demonstrations that you do yourself.”		0.54	
**Aesthetics**							
	Boring color scheme	The color scheme was boring, drab, “depressing,” etc		“Other mental health apps that I’ve seen typically have a little bit better contrast, just things to make the other menu items pop out a little bit more.” “The color could use a little updating.”		0.32	
	Color scheme interferes	The color scheme would interfere with app use or the likelihood of using the app		“Sometimes people’s emotions can be affected by the colors they see and all that. So just brighten it up.”		0.54	
	Color scheme does not interfere	The color scheme would not interfere with app use or the likelihood of using the app		“When you need the help, you need the help. And I don’t think making a whole bunch of pretty colors and all that is really going to change what the app is doing.”		1	
	Amateur graphics	Graphics looked amateur		“People will trust it more if it’s looks more professional.” “In terms of just the buttons, specifically, the little arrow icons, took up half the screen, almost. And it just kind of looked like out of place and too big. And proportions like that matter if you’re going to have a clear and concise experience with the app.”		0.67	
	Outdated graphics	Graphics looked outdated		“It just looked a little outdated, especially the pictures. Like it’s not current.”		0.35	
**Information**							
	Good detail	Liked the level of detail; found it appropriate		“Yeah, I think I think it does a really good job and gives very detailed information.”		0.89	
	Insufficient detail	Insufficient detail; too little detail		No participants made comments about insufficient detail.		1	
	Excessive detail	Felt overwhelmed by information; too much detail		“It’s just like, a lot of information, I guess, and a lot of things to do. I think, personally, I would get a little confused on the app.”		1	
	Learned	Learned new things		“And I wasn’t really like educated on the subject before. So that’s something I did like.” “I learned a lot.”		0.81	
	Credible information	Information seemed credible		“I don’t have any reason to believe that it’s not a credible source.”		0.62	
	Credible source needed	Including information such as references, expert videos, or other ways to make the credibility explicit would be useful		“I feel like there could be some kind of link to information that has credited sources, and not just put those facts up with no way to immediately check the background of it.”		0.51	
**Other**							
	Measurement-based care	Measurement-based care would improve the app (eg, tracking ruminative episodes over time)		“I think the tracking is a good idea, especially to see how much you’ve progressed since starting the app.”		0.06	
	Time gating	Restricting people’s access to parts of the quest based on time (ie, 1 week before you can move on to the next quest) might be frustrating to users		“People do not like time-gated content, especially artificially time gating.”		0.64	

^a^Descriptions and examples of subcategories identified in qualitative focus group feedback.

^b^Cohen κ represents an estimate of interrater reliability for qualitative items.

## Discussion

### Principal Findings

This study used a mixed methods approach and aimed to estimate the initial acceptability of RuminAid by evaluating its user-perceived quality and identifying potential modifications needed. The results of this study suggested that, overall, RuminAid was perceived as “acceptable” to “good” by the focus group participants. In terms of strengths, participants highlighted the information and perceived impact of RuminAid: focus group members reported that the quality of information was quite good and had the potential to help adults who struggle with depression and rumination. In contrast, focus group members expressed concern that poor aesthetics could interfere with users’ desire to continue using the app. Indeed, focus group members highlighted RuminAid’s aesthetics as its primary weakness.

In particular, participants felt that the visual elements of the app—especially the color scheme and graphics—appeared amateur, outdated, and dull. Specific suggestions for improvement included a brighter color scheme, modern aesthetics, and professionally designed graphics. To address these concerns, we hired a graphic designer to redesign all RuminAid screens. The graphic designer was instructed to revise the app screens to look more professional, modern, and colorful, based on participant recommendations. Comparisons of the RuminAid app screens before and after focus group testing are displayed in Figures 1-3 to demonstrate how the data collected in this study directly informed the modifications.

In addition to redesigning the app screens so that they were more aesthetically pleasing, we modified the RuminAid timeline. Although only a minority of focus group participants commented on the “time gating” involved in RuminAid (ie, restricting users from moving from one quest to the next for a period of 1 week), this feedback highlighted potential frustration and loss of interest that might result from this restriction. This is reflected in the literature, which indicates that people use phone apps sporadically [23,88-90], and in lay commentary, which suggested that people dislike apps and games with time-gated content [91]. In addition, attrition tends to be high in mHealth interventions [92-94]. Thus, to prevent frustration and attrition, we reduced RuminAid from a 5-week intervention restricting users to completing 1 quest per week to a 5-day intervention with instructions (but no time gates) to complete 1 quest per day. Notably, we retained the calendar feature for scheduling quests but modified the instructions to fit the revised timeline.

### Limitations

It is possible that demand characteristics and group factors may have played a role in participants reporting that they liked the app in a focus group setting led by a researcher [95]. During the interviews, positive statements about the subjective app quality were made by all but 1 participant. In contrast, the subjective app quality scores obtained via uMARS suggested less satisfaction. However, the uMARS subjective quality scale includes items such as, “would you pay for this app?” The sample of focus group participants consisted entirely of college students, who may be unlikely to pay for any app. More broadly, monetary commitment may not indicate an individual’s true feelings regarding app quality. For example, some individuals may never feel comfortable paying for a smartphone app, regardless of what the app is. Notably, the app quality domain, based on mean scores of engagement, information, functionality, and aesthetics, was more consistent with the feedback provided during focus group interviews. Nevertheless, it is possible that demand characteristics and group factors may have played a role in participants reporting that they liked the app in a focus group setting led by a researcher. In the future, it might be helpful to have the research assistant running the focus group explicitly state that they were not involved in app development. Furthermore, it may be more effective to collect supplemental qualitative feedback via one-on-one interviews to help reduce feedback biases owing to group factors, such as groupthink or reluctance to dissent [96].

Another potential limitation of our study was the use of a sample of college students. We intentionally selected a sample of college students because this population tends to be young and highly digitally literate. As such, college students often have extensive experience using a variety of high-quality smartphone apps with which they could meaningfully compare RuminAid to provide detailed qualitative feedback informed by the current standards of app quality. However, the use of a college student sample may have potentially limited the amount of critical feedback related to app functionality, particularly flow logic. For example, older adults with less digital literacy may have identified more potential difficulties with app navigation and functionality. As we plan for the next stages of this research, it is important to seek feedback from stakeholders of all ages to identify a broader spectrum of potential modifications before making RuminAid available on the public marketplace.

Finally, participants interacted with a story board rather than a beta version of the app itself, and there were limitations to seeking feedback on an app that participants did not have the opportunity to use. Without having first-hand experience of using RuminAid, the participants may have relied on personal biases, expectations, and perceptions to provide feedback on the app’s usability.

### Future Directions

In the next phase of this research, we are most interested in evaluating the core therapeutic content contained within RuminAid as an intervention. As such, we will conduct a pilot trial of RuminAid as an intervention for depression and rumination, which will allow us to examine the app’s feasibility and acceptability and provide initial estimates of its effectiveness among a community sample of adults with depression and rumination. Subsequently, we may conduct a case series study of a beta version of RuminAid. To do so, we may have a community sample of adults with depression install RuminAid and use it for a week before interviewing them individually about their experience. The rich qualitative data collected in such a study would inform any additional modifications made to RuminAid before it is made available to the public.

Once the feasibility, acceptability, and effectiveness of the basic RuminAid approach are established with support for its core therapeutic content, additional features could be incorporated. For example, a substantial percentage of the participants stated that integration of measurement-based care features would be favorable. Measurement-based care has been shown to improve outcomes of treatment for depression [97,98]. Therefore, a longer-term future direction would be to develop measurement-based care features and evaluate whether outcomes are enhanced. Likewise, several participants mentioned wanting a social media component to help them feel more engaged. The incorporation of social media has been shown to improve app engagement [98] and may help to improve long-term app use [99]; therefore, we may also develop and include social features in future iterations of RuminAid.

### Conclusions

RuminAid was intended to address the need for accessible EBT for depression by targeting a key risk factor and maintenance factor—rumination. Although the overwhelming majority of mHealth apps are neither empirically tested nor empirically informed, RuminAid incorporates recent research findings and elements of EBTs for rumination and depression. Should research support its feasibility, acceptability, and effectiveness, RuminAid stands to increase access to treatment for rumination, depression, and their negative sequelae. Importantly, this intervention involved no therapist contact, making it inexpensive and timely, given the recent COVID-19 pandemic and the need for remote service delivery options. The self-guided nature of RuminAid may also minimize stigma-related concerns and "better engage individuals who have limited access to or interest in traditional face-to-face interventions.

In summary, the overall quality and treatment approach used in RuminAid was acceptable to potential users. The feedback obtained in this study directly informed the modifications to RuminAid, and the usability, acceptability, and feasibility of this modified version will be tested in a pilot trial. In terms of its potential impact, RuminAid has broad applications. It is a contactless intervention, based on EBTs, and could reduce the burden on the mental health care system by offsetting care to automated service delivery. If effective, RuminAid could be implemented and tested in a variety of settings such as primary care, behavioral health integrated care, stepped care facilities, or remote care clinics or offered to individuals on clinic waitlists. Potential future augmentations to RuminAid could include peer coaching, therapist coaching, or group facilitation. Consequently, pending empirical evaluation, RuminAid may be an accessible and effective intervention for depression and rumination with positive public health consequences, and its development process could serve as a road map for developing evidence-based mHealth apps and empirically supported mHealth apps.

## Appendix

Multimedia Appendix 1 Psychoeducational content screens. These screens from quests 1, 2, and 5 display psychoeducational content to users to enhance their understanding before skills application. These images are screenshots of RuminAid after the user-centered redesign, which was informed by the results of this study.

Multimedia Appendix 2 Identification and labeling of rumination screens. These screens display the in-app use of skills related to identifying and labeling rumination. These images are screenshots of RuminAid after the user-centered redesign, which was informed by the results of this study.

Multimedia Appendix 3 Alternative behavior screens. These screens from quests 3 and 4 display psychoeducation about alternative behaviors as well as in-app skills use. These images are screenshots of RuminAid after the user-centered redesign, which was informed by the results of this study.

Multimedia Appendix 4 Self-soothing behavior screens. These screens from quest 3 demonstrate how users learn to discriminate between self-soothing and avoidance behaviors. These images are screenshots of RuminAid after the user-centered redesign, which was informed by the results of this study.

Multimedia Appendix 5 Approach behavior screens. These screens from quest 4 display examples of identification of avoidance (left) and generating approach behaviors (right). These images are screenshots of RuminAid after the user-centered redesign, which was informed by the results of this study.

Multimedia Appendix 6 Mindfulness screens. These screens from quest 5 display psychoeducation, in-app skills use, and tips for mindfulness practice. These images are screenshots of RuminAid after the user-centered redesign, which was informed by the results of this study.

Multimedia Appendix 7 Gamified elements screens. These screens display gamified elements of RuminAid, including the quest 1 map in progress and at completion. These images are screenshots of RuminAid after the user-centered redesign, which was informed by the results of this study.

## Abbreviations

BA: behavioral activation
EBT: evidence-based treatment
mHealth: mobile health
PHQ: Patient Health Questionnaire
RFCBT: rumination-focused cognitive behavioral therapy
uMARS: Mobile Application Rating Scale–user version

## Data Availability

All quantitative data sets generated and analyzed during this study are available at Open Science Framework [100]. Full access to the qualitative data collected during focus group feedback sessions is available from the corresponding author upon reasonable request.
