# Supplementary figures and images for: Development of an mHealth App–Based Intervention for Depressive Rumination (RuminAid): Mixed Methods Focus Group Evaluation
Source: JMIR Form Res. 2022 Dec 13;6(12):e40045. doi: 10.2196/40045 (PMC9795400; doi:10.2196/40045)

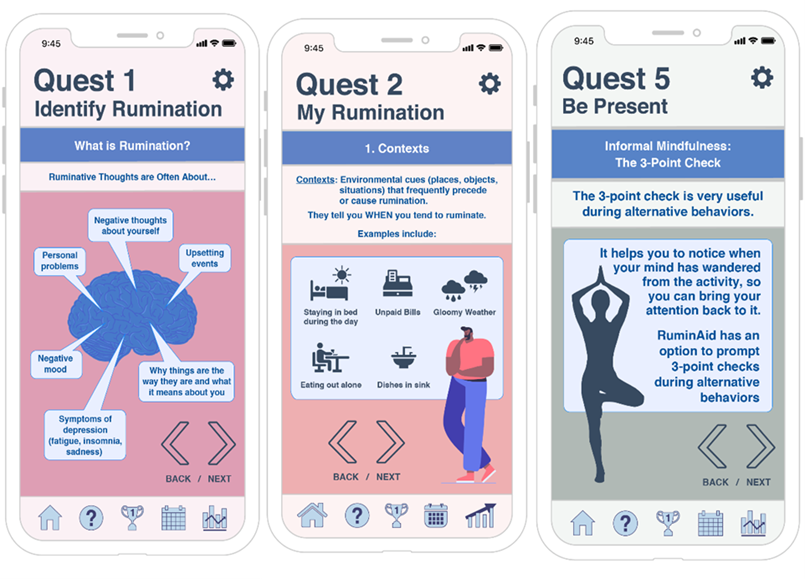

Supplement: Multimedia Appendix 1 [file formative_v6i12e40045_app1.png]

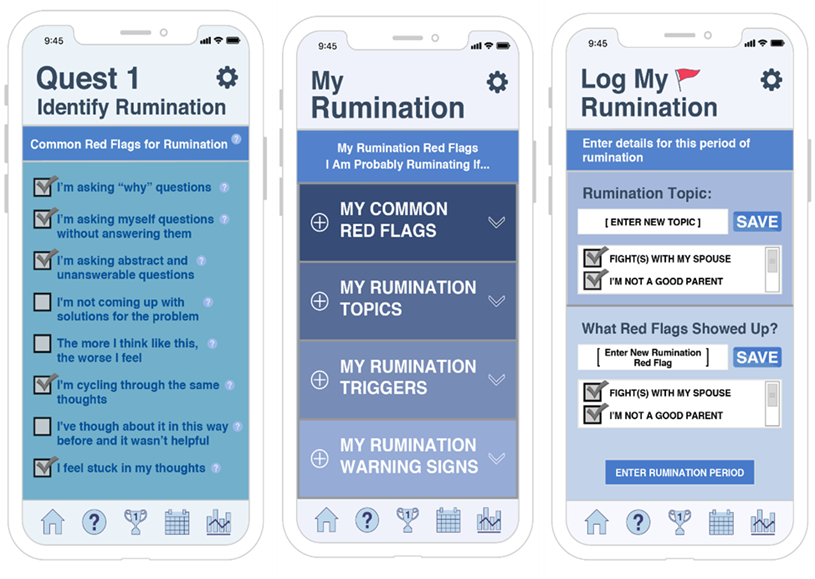

Supplement: Multimedia Appendix 2 [file formative_v6i12e40045_app2.png]

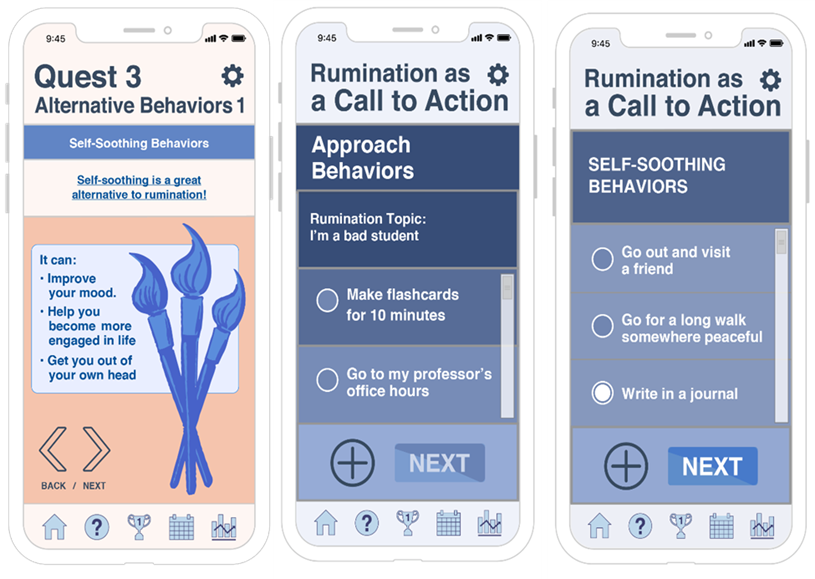

Supplement: Multimedia Appendix 3 [file formative_v6i12e40045_app3.png]

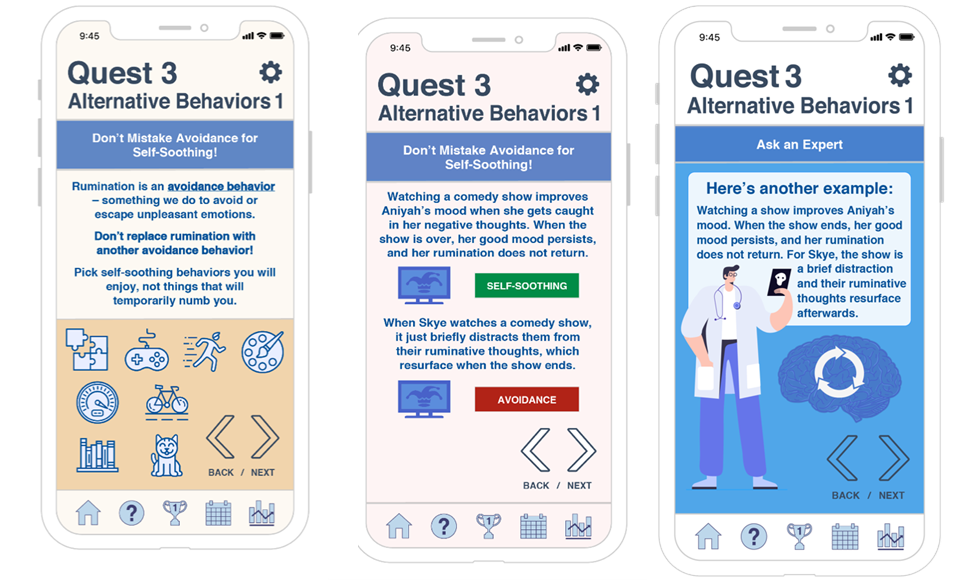

Supplement: Multimedia Appendix 4 [file formative_v6i12e40045_app4.png]

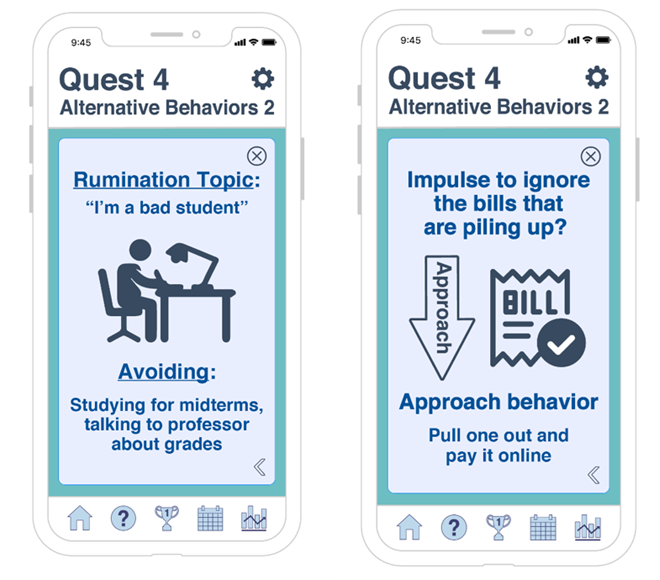

Supplement: Multimedia Appendix 5 [file formative_v6i12e40045_app5.png]

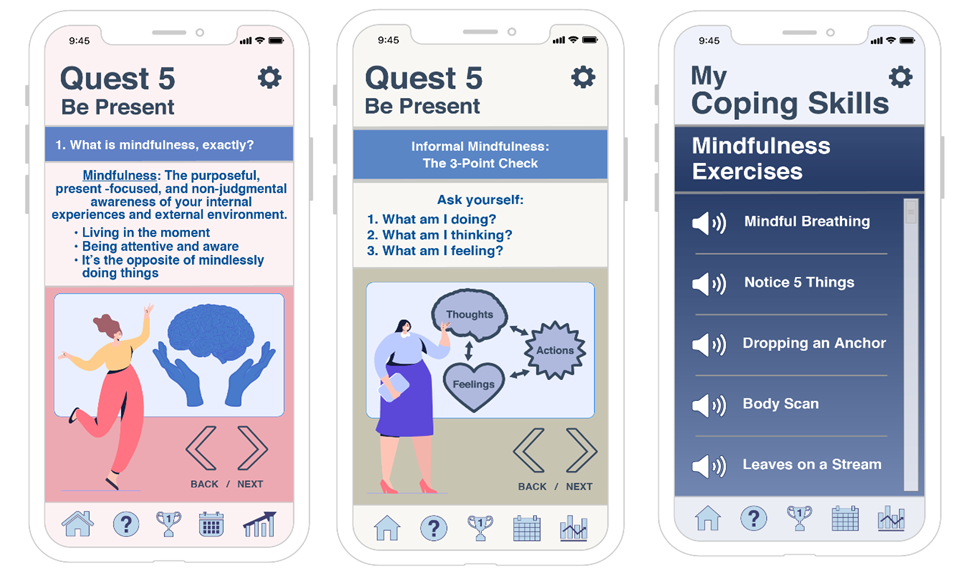

Supplement: Multimedia Appendix 6 [file formative_v6i12e40045_app6.png]

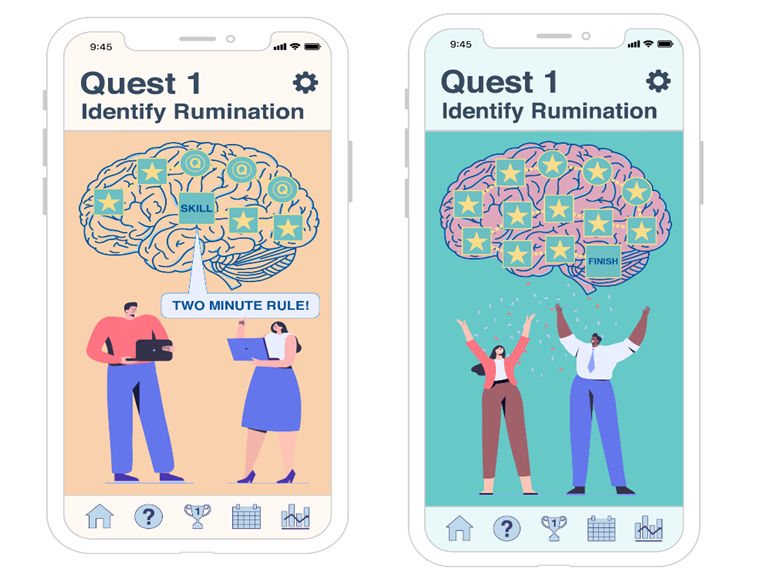

Supplement: Multimedia Appendix 7 [file formative_v6i12e40045_app7.png]
